# Microfluidic investigation of synergistic mechanisms of microsphere-microbial compound system for enhanced oil recovery

**DOI:** 10.1038/s41598-026-44131-1

**Published:** 2026-03-19

**Authors:** Hua Li, Weiyao Zhu, Zhiyong Song, Zhen Chen, Bingbing Li

**Affiliations:** 1 School of Mechanics and Engineering, Liaoning Technical University, Fuxin, 123000 China; 2 School of Civil and Resource Engineering, University of Science and Technology Beijing, Beijing, 100083 China; 3 School of Mechanical Engineering, Liaoning Technical University, Fuxin, 123000 China; 4 School of Safety Science and Engineering, Henan Polytechnic University, Jiaozuo, 454000 China

**Keywords:** Compound system agent, Enhanced oil recovery, Micro-PIV, Oil displacement mechanism, Synergistic effect, Engineering, Microbiology

## Abstract

Field tests have shown that microsphere-microbial compound flooding improves the oil recovery factor. The compound system consists of surfactant-modified polyacrylamide nanospheres (initial diameter is 150 nm) and an enriched indigenous microbial consortium. However, the detailed mechanisms remain unclear. To address this gap, this study employs microscopic visualization and particle image velocimetry (PIV) to compare the compound system with single-component microsphere and microbial flooding. Results indicate that the compound system achieves recovery factors 7.26% and 4.54% higher than microbial and microsphere flooding, respectively, confirming a synergistic effect. Its displacement efficiency and sweep effectiveness are also superior. Mechanistic analysis reveals a stage-dependent advantage. During injection, surfactants released from microspheres alter wettability. In the shut-in stage, microbial metabolites further enhance displacement, producing dual benefits. Under dynamic conditions, blocking performance was quantified. PIV results demonstrate that the compound system significantly reduces velocity heterogeneity of the flow field. Single‑channel velocimetry reveals smaller velocity fluctuations (< 15%), indicating stronger shear stability and improved plugging performance relative to either agent alone. Microscopic observations further reveal aggregate structures in which the microsphere skeleton supports microbial adhesion. This enables stable spatial distribution under high shear and effective blockage of dominant channels. By integrating quantitative evidence from visualization and velocimetry, this study provides a useful approach for evaluating compound flooding performance aimed at improving oil recovery for mid-low permeability reservoirs.

## Introduction

In recent years, the accelerated global economic development and industrialization have led to a continuous increase in crude oil demand. The crude oil consumption is projected to remain at an exceptionally high level in the foreseeable future^1^. China’s proven oil reservoirs are experiencing severe high water cut issues^2^, and the recovery factor stagnates at 25% to 50%^3^. Therefore, developing efficient technologies to achieve high recovery factors adapted to China’s complex reservoirs has become the key to ensuring energy security^4–6^. Traditional chemical flooding agents (e.g., polymers, surfactants) are often ineffective in mobilizing residual oil in high-water-cut and highly heterogeneous reservoirs^7^. Consequently, microsphere and microbial flooding have gained attention for their superior injectivity, profile control, and low cost^8,9^. Moreover, the limitations of these individual agents have prompted research into synergistic microsphere-microbial compound systems (2M compound systems)^10–12^. These systems aim to improve recovery and stabilize production in oilfields.

Microspheres are micron-sized spherical particles that selectively block high-permeability layers through water-induced swelling. By redirecting displacement fluids to low-permeability zones, they mobilize residual oil and enhance sweep efficiency^13^, potentially increasing recovery factors by 6–17%^8,14^. Microspheres also demonstrate advantages in improving the mobility ratio and regulating the displacement process^15,16^. However, chemical effects of microspheres are of limited duration, restricting sustained enhancement of crude oil fluidity and residual oil recovery^17^. Therefore, combining microspheres with other agents has become an important research direction.

Microbial oil displacement relies on the growth and metabolism of specific microbial strains in reservoirs. These strains produce metabolites—primarily surfactants—that mobilize residual oil through improved wettability, crude oil emulsification, and selective migration^18–21^. This approach has demonstrated effectiveness in some high-water-cut mature oilfields^22–24^. Nonetheless, microbial flooding faces key challenges. Complex migration in porous media hinders uniform distribution, reducing effectiveness in certain reservoirs^25^. Additionally, its primary mechanisms—wettability alteration and emulsification—are less effective in weakening dominant channels and sustaining long-term sweep efficiency^26^.

In view of the limitations of microbial and microsphere flooding in single applications, recent studies have focused on combining them into a 2M compound flooding system to exploit complementary advantages. For instance, Yang et al.^27,28^ demonstrated field feasibility and notable recovery gains. However, further research remains limited, with few published cases suggesting potential applicability primarily in mid-low permeability reservoirs. Other particle-bioactive systems—such as bio-nanocomposite fluids^29^, SiO_2_-biosurfactant nanofluids^30,31^, microbially synthesized silver nanoparticles^32^, and graphene-biosurfactant systems^33^—have demonstrated synergistic flooding via interfacial-tension reduction, wettability alteration, or enhanced emulsification. These findings offer mechanistic insight relevant to polymer microsphere-microbial systems. However, the polymer microsphere-microbial system requires systematic study of its specific advantages. In particular, the coupling between interfacial phenomena and micromechanical mechanisms remains underexplored, hindering broader application.

Although limited field and mine reports suggest practical potential for similar composite systems, mechanistic understanding remains limited. This study aims to achieve two objectives: (1) to elucidate the microscopic synergistic mechanisms of the microsphere–microbial compound system through micromodel experiments, and (2) to quantify the associated incremental oil recovery under representative laboratory conditions.

To rigorously evaluate the efficacy of the compound system, it is essential to benchmark it against single-component systems. Single microsphere flooding often encounters challenges, such as migration channeling. Similarly, single microbial flooding is constrained by its cell concentration and plugging strength. By comparing the compound system with these single-component baselines, this study aims to determine whether interactions between microspheres and microbes generate a synergistic effect that specifically addresses the limitations of the individual agents. This investigation involves three primary approaches: (1) Static characterization using optical microscopy to examine the microstructure of microspheres and microbial cells. (2) Microscopic visualization of oil displacement to characterize flow behavior and displacement modes induced by the compound system. (3) Particle Image Velocimetry (PIV) to obtain quantitative hydrodynamic data and reveal potential synergies. Ultimately, this research seeks to elucidate the mechanisms of synergistic action between microspheres and microorganisms, thereby providing theoretical and experimental support for enhanced oil recovery strategies.

## Experimental materials and methods

### Materials

A simulated oil (50 mPa s at 60 °C) was prepared by diluting crude oil from a Chinese oilfield with kerosene. Formation water from the same oilfield (salinity: 3400 mg/L) was used as the aqueous phase.

The three oil displacement agents used in the experiment were formulated as follows: (1) Microsphere solution (WQ_100_): WQ_100_ is one of the most widely applied microsphere dispersion systems for oil displacement. It was prepared via inverse emulsion polymerization by mixing polymer chains (mainly acrylamide) with surfactants Span 80 and Tween 80 to form surfactant-encapsulated polymer particles. The microspheres had an average particle size of approximately 150 nm and exhibited significant swelling upon hydration, with a swelling ratio of 5.16. The solution was prepared at a volumetric concentration of 0.2%, a standard formulation widely adopted in Chinese oilfields. (2) Microbial solution: An indigenous microbial consortium was enriched from the produced water of Shengli Oilfield and prepared at a volumetric concentration of 6%. The cell concentration after enrichment culture was 1 × 10⁸ cells/mL. The activation medium was composed of glucose (40 g/L), sodium nitrate (4 g/L), KH_2_PO_4_ (4.1 g/L), Na_2_HPO_4_·12H_2_O (14.3 g/L), and MgSO_4_·7 H₂O (0.2 g/L), with pH adjusted to 7.0. (3) Microbial-microsphere compound system: The compound system was formulated by combining 6% microbial culture solution with 0.1% microsphere dispersion (WQ_100_). This formulation demonstrated good synergistic effects in terms of stability and oil displacement performance and was therefore selected as the standard formulation for all subsequent experiments.

After formulation, the three agent solutions were transferred into clean flasks and cultivated for 72 h at 60 °C and 120 rpm. Samples were observed every 12 h using an AFT-DC200 metallographic microscope. Microbial cells were stained with methylene blue to differentiate them from microspheres.

### Process of microscopic visualization flooding experiment

#### Microscopic visualization chip model

**Fig. 1:**
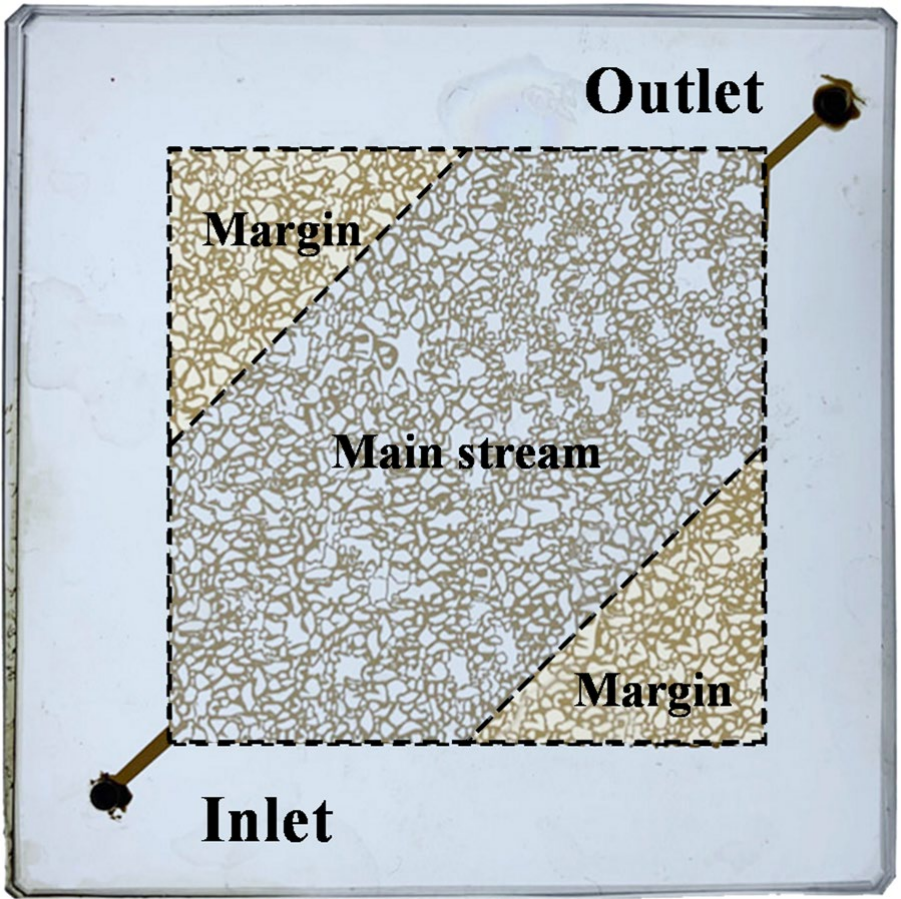
Area division diagram of the microfluidic model.

Oil-water interfacial dynamics were observed in a microscopic visualization chip to evaluate different displacement agents. The micromodel geometry was traced from a real core slice collected from Shengli Oilfield and converted into a photomask. The glass-based chip was then fabricated via photolithography and chemical etching on a glass substrate. Image analysis confirmed an areal porosity of 18.55% and a pore-throat size distribution matching the parent core, with pore diameters ranging from 20 to 100 μm. The crude oil aging method was applied to render the wettability of the model closer to the real state, and the microscopic model of saturated oil was aged for 2 months at 80 °C. The model was 6.35 cm × 6.35 cm in size, the pore network area was 4 cm × 4 cm, the pore plane diameter ranged from 20 μm to 100 μm, and the etch depth was 30 μm. The model was divided into the main streams and marginal areas to facilitate quantitative analysis of different areas (Fig. 1).

#### Microscopic visualization experiment for oil displacement

**Fig. 2:**
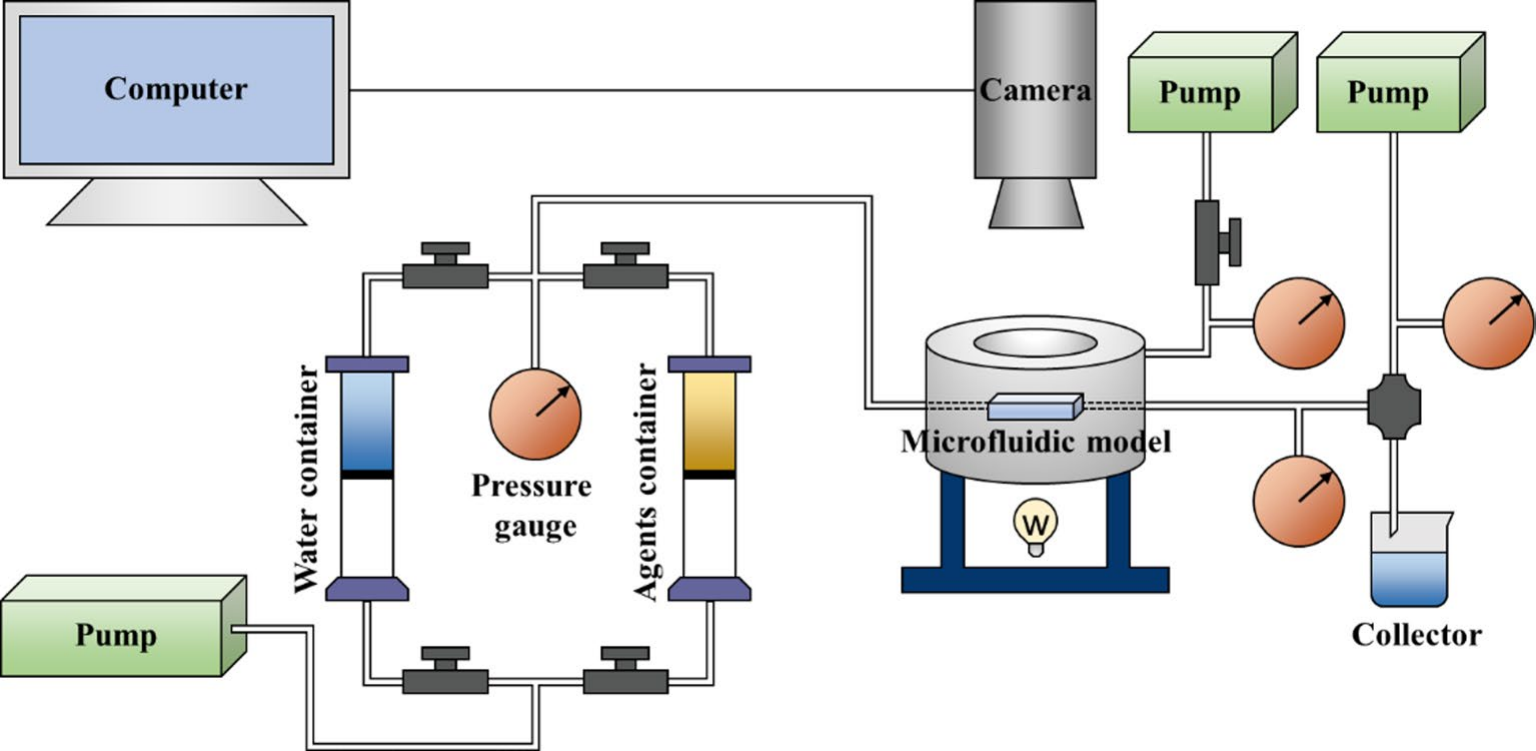
High-temperature, high-pressure visualization experiment device.

In the experiment, an independently developed microscopic visualization oil displacement device was used, which included injection pumps, model holders, a stereomicroscope, planar light sources, and a computer monitoring system (Fig. 2). The experimental procedure was as follows: (1) the model was evacuated and saturated with formation water; (2) the model was saturated with simulated oil by displacing the water until no further effluent was observed; (3) water flooding was carried out at a constant rate until the outlet water cut reached 98%; (4) the displacement agent was injected at 3 pore volumes (3 PV); and (5) a subsequent water flooding (i.e. post-water flooding) was performed until the residual‑oil pattern stabilized. The injection rates in all experiments were 8 µL/min. Oil-water distributions and dynamic changes were recorded using microscope.

Images were analyzed using Python programs; phases were separated by grayscale thresholding, and global and local pore oil saturations were computed from oil‑phase pixel proportion. The specific calculation formula is given by

1 $$\begin{array}{*{20}c} {S_{o} = A_{{oil}} /A_{{total}} \times 100\% ~} \\ \end{array}$$

where $S_{o}$ is the oil phase saturation expressed as a percentage; and $A_{{oil}}$ and $A_{{total}}$ are the dimensionless numbers of the oil phase and effective pore pixels identified in the images, respectively.

### Microscopic flow field research device and method

**Fig. 3:**
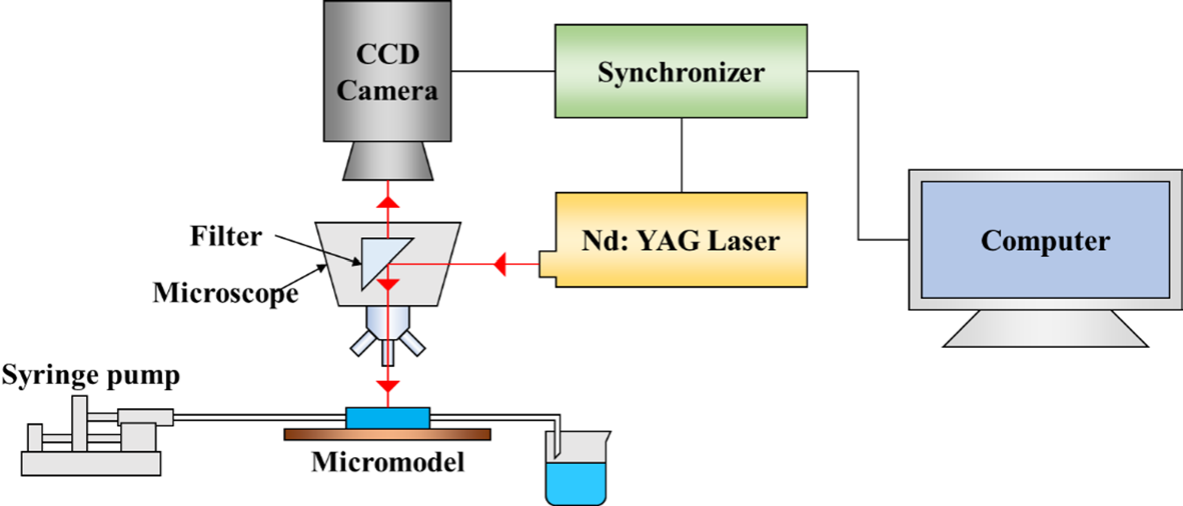
Velocity field test schematic diagram.

The velocity distribution in the porous medium was captured using a Micro‑PIV system (LaVision, Germany) comprising a 200 mJ Nd: YAG laser (532 nm, 15 Hz), a 12‑bit CCD camera with a 10× objective (NA = 0.3), and a microfluidic displacement device (Fig. 3). Fluorescent 1 μm microspheres (0.005% seeding) were sonicated before use. Injection pressure was measured by an electronic manometer (0–50 kPa, Senex, USA). The experimental procedure was as follows: (1) model evacuated and saturated with formation water; (2) formation water injected for 3 PV; (3) displacement agent injected for 3 PV; (4) formation water re‑injected for 3 PV. The injection rates in all experiments were 8 µL/min. Stable flow fields (Fig. 1) in main streams and the margin area at each stage were recorded and computed in DaVis, and the average velocity was calculated by

2 $$v_{{{\mathrm{avg}}}} = \mathop \sum \limits_{{i = 1}}^{N} \frac{{v_{i} }}{N}~$$

where $v_{{{\mathrm{avg}}}}$ is the average velocity at the observation point (m/s); ${v_{i} }$ is the velocity magnitude of each vector in the observation field of view (m/s); and $N$ is the dimensionless number of velocity vectors in the observation field of view.

## Results

### Agglomeration of the microbial-microsphere compound system

**Fig. 4:**
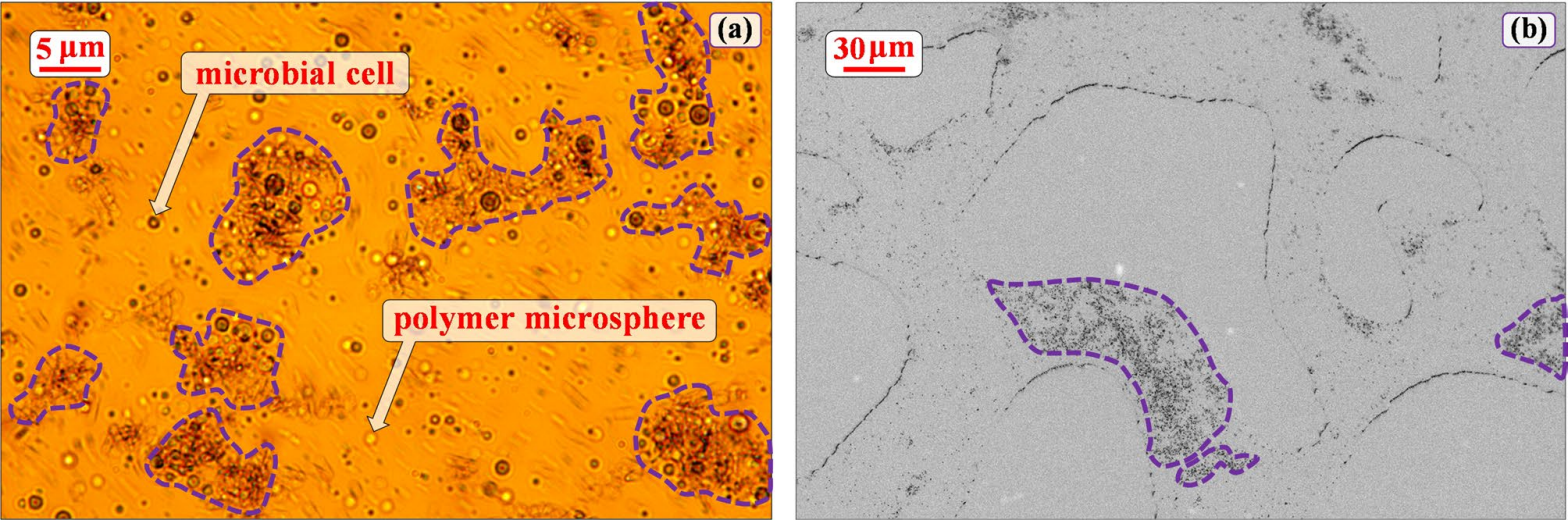
(a) Microscopic observation after incubation with the 2M compound system: The dark colored particles are microbial cells, the light colored particles are polymer microspheres, and the purple dotted line defines the aggregate structures; (b) The aggregate structures within the microfluidic chip during the PIV experiments, and the purple dotted line defines the aggregate structures.

After 72 h of co-cultivation, the microscopic observations (Fig. 4(a)) showed that most microbial cells and microspheres formed aggregates (with a size distribution of 5 to 20 μm), and a few cells and microspheres were evenly and discretely distributed in the medium. Figure 4(b) presents a representative microscopic image within the microfluidic chip during PIV experiments.

### Enhanced oil recovery factor by the microbial-microsphere compound system

**Fig. 5:**
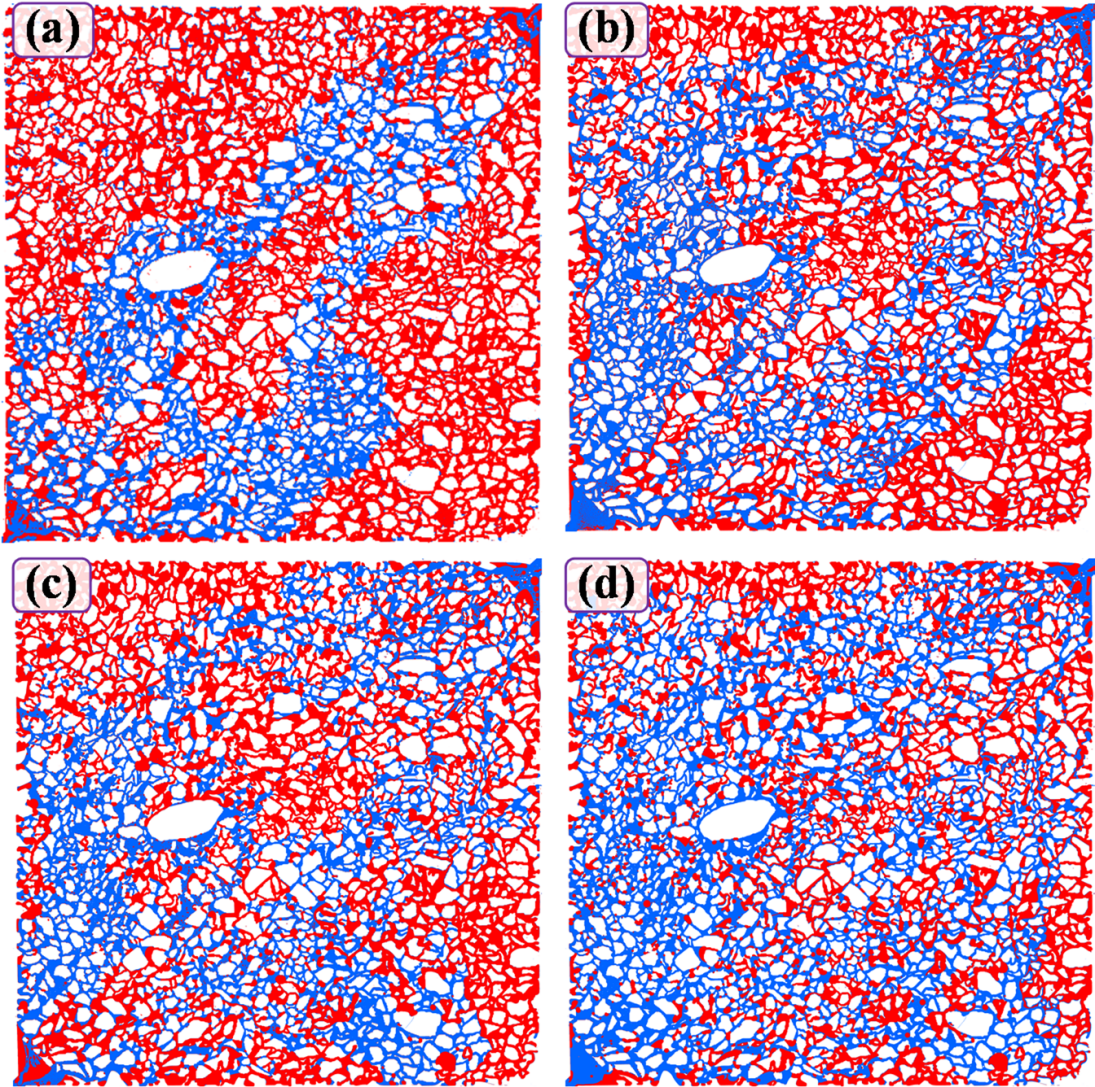
Residual oil distribution pattern in the entire pore network following treatment with the compound system (Red marks crude oil, blue marks displacement fluid, and white marks solid matrix; same below): (a) after primary water flooding; (b) after 2M compound system flooding; (c) static incubation 20d; (d) after post-water flooding.

The changes in the residual oil in the micropores were recorded after the primary water flooding, the agents flooding, the 20-day static incubation, and the post-water flooding. Only images from the 2M compound flooding system are presented because this study primarily focuses on this agent; however, similar qualitative patterns were observed for other displacement agents. It can be seen that the main flow channel from the inlet to the outlet shows obvious fingering after the primary water flooding, and the residual oil saturation in the margin areas is close to 100% (Fig. 5(a)). Injection of the compound system significantly reduced the residual oil saturation in the margin areas (Fig. 5(b)); during 20 days of static incubation, the mobilized region further expanded (Fig. 5(c)); post-water flooding caused a marked increase in the swept area (Fig. 5(d)).

The quantitative recovery factors from microscopic visualization flooding experiments with different agents are presented in Table 1. The 2M compound system achieved the highest overall recovery factor, exceeding the microbial and microsphere flooding systems by 7.26% and 4.54%, respectively (Table 1). Regional analysis reveals more pronounced improvements in the main flow channel (5.71% and 5.98% higher than microbial and microsphere flooding, respectively) and particularly in the margin areas (11.15% and 1.54% higher, respectively), demonstrating superior displacement efficiency across multiple regions.

**Table 1:** Oil saturation and recovery factor enhancement results with each agent.

		After water flooding, *S*_o_/%	After agent flooding, *S*_o_/%	After 2nd water flooding, *S*_o_/%	Enhanced oil recovery/%
Microbial solution	Main stream	61.07 ± 3.45	55.99 ± 4.56	44.68 ± 1.05	16.39
	Margin	97.41 ± 1.89	94.51 ± 2.34	58.43 ± 2.89	38.98
	Overall	70.01 ± 3.12	65.62 ± 3.78	48.12 ± 2.65	21.89
Microsphere- based agent	Main stream	61.76 ± 4.44	47.57 ± 3.21	45.14 ± 1.03	16.62
	Margin	95.08 ± 2.18	82.09 ± 1.82	46.48 ± 2.37	48.60
	Overall	70.09 ± 2.88	56.20 ± 1.46	45.48 ± 2.19	24.61
2M compound system	Main stream	62.44 ± 4.77	46.82 ± 1.56	40.34 ± 2.73	22.10
	Margin	94.74 ± 3.45	80.68 ± 3.67	44.61 ± 2.50	50.13
	Overall	70.52 ± 1.23	55.29 ± 1.74	41.36 ± 1.51	29.16

The “±” values represent the standard deviation for 3 samples.

### Wetting angle variation in microbial and compound systems

**Fig. 6:**
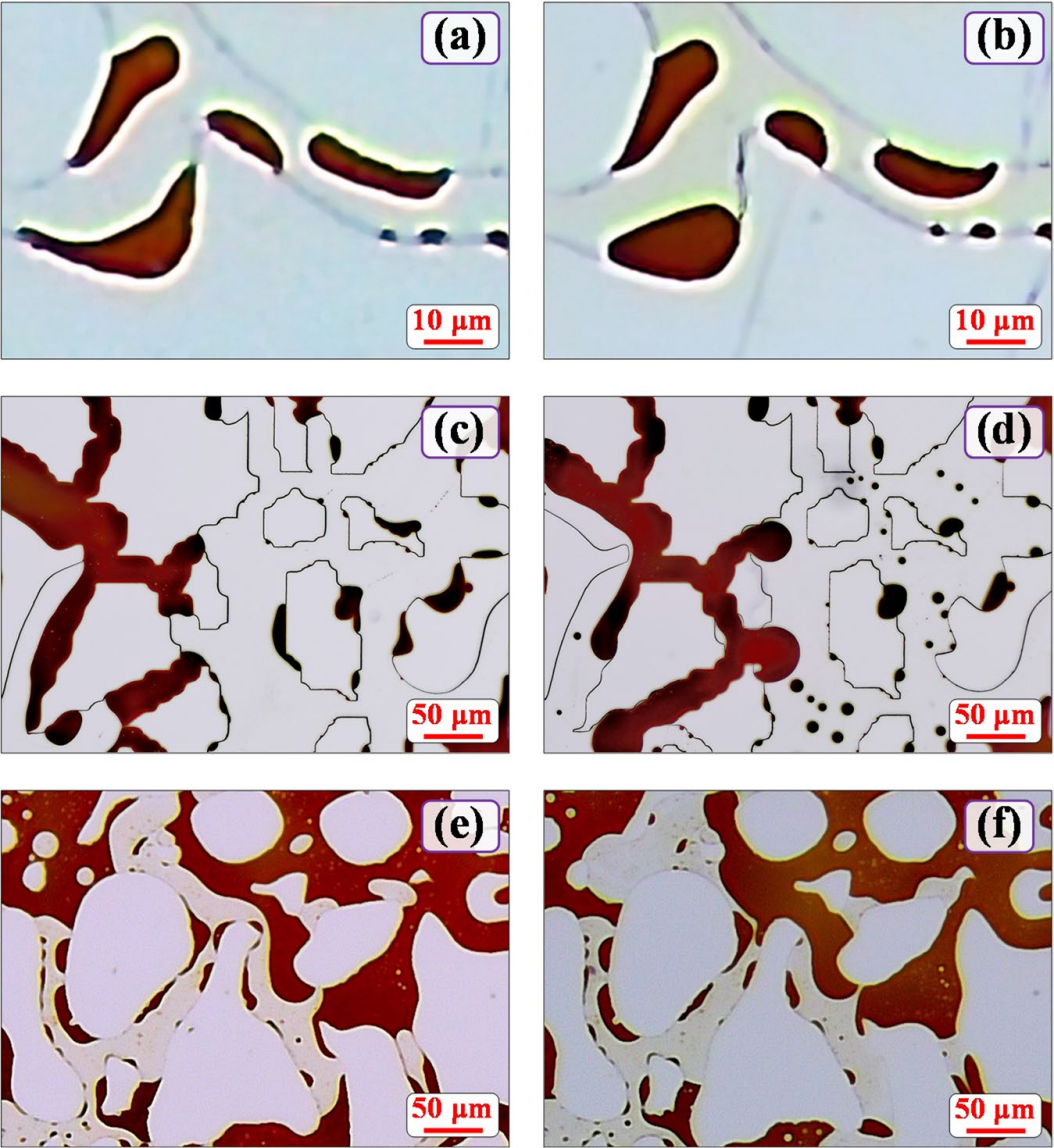
Microscopic observations of wettability changes induced by the two agents: (a) after microbial solution injection; (b) after static incubation 5d; (c) after 2M compound system injection; (d) after static incubation 5d; (e) after microspheres solution injection; (f) after static incubation 5d.

During the microscopic visualization experiment, the residual oil wetting angle in the pore network increased significantly after injecting the microbial solution and the compound system (Fig. 6). Microscopic images captured from the inlet, middle, and outlet of the main flow channel were analyzed, with average wetting angle values from 30 random sample points at each location presented in Table 2. The wetting-angle differences between the microbial and compound systems at each location were about 10°. For comprehensive analysis, Fig. 6; Table 2 also include results for the single‑microsphere system, which consistently showed negligible wettability alteration, reaffirming that microspheres do not continuously change the wetting properties.

**Table 2:** Residual oil Wettability angle changes under the action of the compound system. The “±” values represent the standard deviation for 30 samples.

		Agent flooding	Static incubation 5 d	Angle changing
Microbial solution	Main channel inlet	42° ± 10.5°	109° ± 12.4°	+ 67°
	Main channel center	47° ± 12.8°	102° ± 9.6°	+ 55°
	Main channel outlet	41° ± 2.2°	69° ± 11.9°	+ 28°
2M Compound system	Main channel inlet	47° ± 11.5°	124° ± 13.5°	+ 77°
	Main channel center	45° ± 12.4°	110° ± 15.1°	+ 65°
	Main channel outlet	42° ± 13.6°	80° ± 16.7°	+ 38°
Microspheres solution	Main channel inlet	53° ± 9.5°	54° ± 8.8°	+ 1°
	Main channel center	50° ± 10.2°	51° ± 11.4°	+ 1°
	Main channel outlet	47° ± 8.4°	49° ± 9.2°	+ 2°

### Microscopic velocity field characteristics

**Fig. 7:**
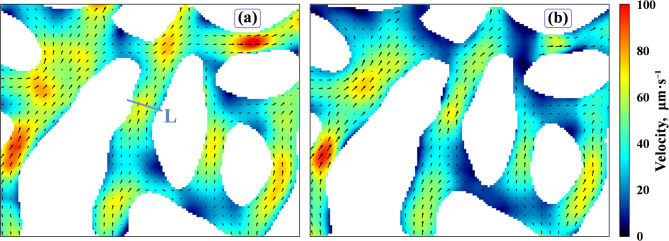
Velocity distribution cloud diagram with the compound system: (a) after 2M compound system flooding; (b) after post-water flooding.

Figure 7 shows the microscopic velocity distributions in the inlet area of the main flow channel during compound system injection and subsequent waterflooding. During compound system injection, the fluid exhibits rapid flow through both large and small pores. In contrast, following waterflooding, the overall velocity at the same location decreased significantly.

Table 3 shows the pore-scale velocity distribution following displacements with water and the three agents. $R_{{\mathrm{m}}}$ is defined as the velocity ratio of the main stream to the margin areas, and is used to evaluate the uniformity of the microscopic flow field, with a lower value indicating more uniform flow^34^. As shown in Table 3, the 2M compound system exhibited lower $R_{{\mathrm{m}}}$ during both the flooding stage (1.88) and the post-waterflooding stage (1.70) than the other two agents, indicating a more uniform velocity distribution and a sustained advantage after waterflooding.

**Table 3:** Average velocity distribution (Unit: µm/s) and *R*_m_ with different agents flooding (The transition areas refer to the demarcation lines between the main stream and the margin areas).

		Main stream	Transition	Margin	*R*_m_	Injection pressure (kPa)
Water flooding		12.8	10.4	6.3	2.03	3.05
Microbial	Agent flooding	23.13	15.36	8.79	2.63	5.83
	Post-water flooding	22.63	13.86	8.61	2.63	5.60
Microsphere	Agent flooding	20.16	15.35	12.6	1.60	7.41
	Post-water flooding	16.54	15.09	5.12	3.23	6.69
2M compound system	Agent flooding	36.26	23.09	19.33	1.88	9.98
	Post-water flooding	28.56	13.53	16.84	1.70	8.34

### Pore-scale velocity profile characteristics with the blocking of compound system agglomerations

**Fig. 8:**
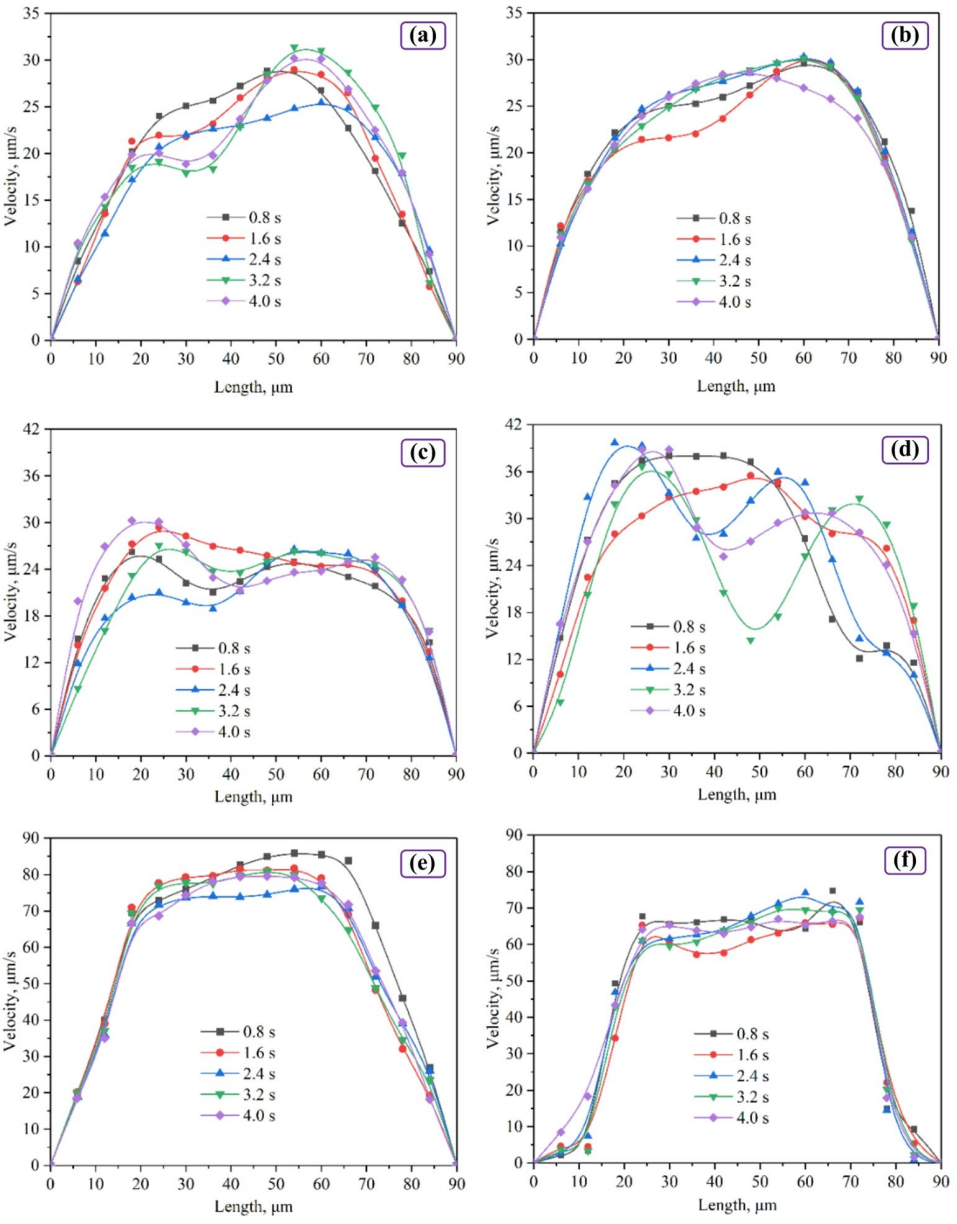
Velocity profile during microsphere injection: (a) microbial solution flooding; (b) post-water flooding; (c) microsphere solution flooding; (d) post-water flooding; (e) 2M compound system flooding; (f) post-water flooding.

Figure 8 shows the pore cross-section velocity profiles in the main stream area (line “L” in Fig. 7) for three cases: microbial (subplots a, b), microsphere (subplots (c, d), and 2M compound system (subplots (e, f), during both agent flooding and post-water flooding. For the microbial solution, fluctuations during injection were below 15 μm/s, with no significant changes, and the effective flow radius was about 82 μm. The effective flow radius is defined as the radial range where velocity exceeds 20% of the maximum value^34^, which can indicate the agent plugging performance. And the fluctuations are defined as the maximum temporal velocity change observed for any point in pore within the effective flow radius during a measurement cycle. This metric captures the transient mechanical stability of plugging structures—more critical for assessing blockage integrity than standard deviation. The microsphere solution exhibited a platform-like profile with fluctuations below 10 μm/s during injection, but showed pronounced fluctuations with peak shifts and concave patterns later, giving a effective flow radius of about 83 μm. The 2M compound system showed a clear platform and small fluctuations (below 15 μm/s) during flooding; post-water flooding reduced both the platform maximum and pore velocities, decreasing the radius to 66 μm.

## Discussion

### The comparatively better recovery factor demonstrates the synergistic advantages of the compound system

Microspheres and microbials can improve the recovery factor when applied alone. However, the former is prone to blocking in the near-well area. The latter struggles to achieve even distribution, and its residual oil mobilization effect is limited (see Introduction). To overcome the limitations of both, this study constructs a microsphere-microbial compound system and evaluates its synergistic potential through a microscopic experimental system.

Results from multiple microscopic visualization experiments show that, with a reduced microsphere dosage (50% of the single microsphere solution), the 2M compound system achieved recovery factors 7.26% and 4.54% higher on average than microbial and microsphere solutions, respectively (Table 1). Notably, the superior performance of the 0.1% composite system compared to the 0.2% single microsphere system demonstrates that microorganisms effectively compensate for the reduced microsphere dosage, confirming true synergistic interaction. This consistent trend aligns with observations by Han et al.^28^ in multiple core experiments, suggesting that the compound system not only further improves the recovery factor but also offers benefits of cost reduction and enhanced efficiency through reduced microsphere dosage.

Despite the compound system’s enhanced efficiency, elucidating its dominant mechanisms is crucial to provide a theoretical basis for optimization^35,36^. As the recovery factor is determined by displacement efficiency and sweep efficiency^37^, this study utilized microscopic visualization experiments to evaluate the agents’ performance in these two aspects.

Specifically, the compound system’s enhanced performance results from the synergistic interplay of microspheres, which primarily improve macroscopic sweep efficiency via fluid diversion, and microbes, which contribute to microscopic displacement efficiency through biochemical effects. This allows for a two‑stage improvement in oil recovery.

### The relay effect of the surfactants in the microsphere-microbial compound system leads to higher displacement efficiency

Oil displacement efficiency, primarily driven by an agent’s ability to mobilize residual oil through wettability alteration^38^, is quantifiable by the mainstream recovery factor within a microscopic model^39^. According to Table 1, after injection the oil displacement efficiency ranks: compound system > microbial system > polymer microsphere system. The compound system’s advantage over the polymer microsphere system primarily stems from the synergistic chemical and physical effects of the polymer microspheres during injection. The surfactant encapsulated within the microsphere system, despite its limited and non-renewable nature (< 15% by mass)^40^, rapidly lowers interfacial tension and improves wettability. However, as shown in Table 2, microspheres cannot continuously release surfactants during static incubation. Additionally, mainstream microspheres block larger channels and increase local flow velocity, thereby mobilizing residual oil that would otherwise be difficult to dislodge^14^. At the injection stage, the compound system reduced mainstream oil saturation by 15.62%, which was close to the 14.19% observed for the polymer microsphere system and substantially higher than the 5.08% for the microbial solution, consistent with a microsphere-dominated mechanism.

After incubation, post-water flooding, the reductions in mainstream oil saturation (relative to the agent injection stage) were: 11.31% for the microbial solution, 6.48% for the compound system, and 2.43% for the polymer microsphere system. The weak contribution of polymer microspheres at this stage is expected because they release surfactant only during injection and cannot sustain it during incubation^41^, while microorganisms continuously produce surfactants. The microbial surfactant production is delayed but persistent^26^; therefore, the 2M compound system’s incubation‑stage performance is mainly attributable to microbial effects. However, the smaller incubation‑stage reduction observed for the compound system compared with microbial solution, despite their similar wettability indicators (Table 2), suggests additional limitations: (1) some oil requiring wettability change was already mobilized during compound system injection; (2) microsphere-microbe aggregates trap microbes, reducing chemotaxis and distribution^42^; (3) the contribution of wettability improvement to recovery has an upper limit^43^. Therefore, considering the substantial oil reduction during the injection phase, pre-mobilization of oil emerges as the dominant factor limiting further reduction during incubation. Nevertheless, the compound system achieves a higher final cumulative recovery, attributable to the polymer microspheres’ first-mover advantage during injection and the continuous production of microbial metabolites during incubation. This temporal complementarity, characterized by a pronounced microsphere effect during injection followed by sustained microbial activity during incubation, results in a two-stage improvement in oil displacement efficiency.

This study employed a 20-day shut-in period to ensure robust mechanistic verification, a duration consistent with those commonly reported in similar laboratory investigations^44,45^. However, microscopic imaging revealed minimal further change beyond 10 days, suggesting that shorter shut-in intervals could suffice for mechanistic observation while enhancing laboratory efficiency. For field application, optimal shut-in times are typically longer than those in laboratory settings, primarily due to scale-up effects, slower mass transfer, and reservoir heterogeneity. Therefore, optimizing shut-in timing to balance mechanistic validation, operational efficiency, and field-scale applicability will be a priority for future work.

### The long-term stable blocking of dominant channels is a key factor for the better sweeping effect of the compound system

Sweep efficiency reflects a displacement agent’s capacity to suppress fingering and increase the swept volume^46^, and can be evaluated at the pore scale by margin recovery^47^. Table 1 ranks the sweeping capacity as: compound system > microsphere solution > microbial solution. To elucidate the mechanistic basis for this ranking, dynamic fluid-agent interactions warrant analysis using PIV-derived flow fields^48,49^.

PIV statistics (Table 3) delineate distinct velocity distributions during injection and post-water flooding stages, correlating with sweep expansion. For the microbial solution, mainstream velocities (23.13 and 22.63 μm/s) substantially exceeded margin velocities (8.79 and 8.61 μm/s), resulting in an *R*_m_ (main-to-margin velocity ratio) > 2.6, thereby indicating a persistent dominant channel and limited diversion. The microsphere solution exhibited an injection‑stage *R*_m_ of 1.60 (indicative of initial blocking) that increased to 3.23 after water flooding; margin velocity decreased by 59%, consistent with washout of the blocking structure and reduced diversion. By contrast, the compound system maintained low *R*_m_ values (1.88 and 1.70) with only 13% decline in margin velocity, indicating sustained supply to margin regions while the main channel was plugged. The injection pressures for the 2M compound system were the highest during both the injection and post-water flooding stages (Table 3), also indicating that the 2M compound system provides effective and stable channel plugging.

Absolute velocity measurements corroborate genuine flow redistribution. In the post-water flooding stage, the compound system’s margin velocity (16.84 μm/s) exceeded those of the microsphere solution (5.12 μm/s) and microbial solution (8.61 μm/s) by factors of 3.3 and 1.95, respectively, while the mainstream velocity decreased by 21% (from 36.26 to 28.56 μm/s). These observations indicate that sweep expansion arises from volumetric flow reallocation rather than from a superficial increase in *R*_m_ caused solely by main‑channel deceleration.

Radial velocity profiles provide additional mechanistic insight. A reduced effective flow radius denotes channel blocking^34^, and small velocity fluctuations imply stable blocking structures. The microbial solution showed small fluctuations but a large effective radius (82 μm), indicating no sustained blocking; biopolymers are also known to be susceptible to shear degradation^50^. The microsphere solution formed an initial blocking but later exhibited larger fluctuations and an increased effective radius (83 μm), suggesting structural disintegration under shear. In contrast, the compound system displayed stable fluctuations (< 15 μm/s), substantially lower than other agents, a reduced effective radius (66 μm) and broader, persistent platform features—behavior consistent with microsphere-microbe agglomeration that stabilizes spatial distribution under shear^51^. These lower fluctuations provide strong evidence for superior mechanical stability of the compound system. Collectively, these results are consistent with agglomeration contributing to the compound system’s enhanced shear resistance. Thus, agglomeration within the compound system may be a key mechanism underlying its enhanced sweep expansion.

While microscopic observations and PIV results provide indirect evidence for aggregate stability, direct characterization of formation mechanisms (e.g., electrostatic interactions, hydrophobicity) and mechanical properties under shear remain for future investigation using techniques such as zeta potential measurements and shear rheology tests.

While the 2D model used in this study simplifies 3D reservoir connectivity, the fundamental mechanisms (such as selective bypassing, capillary trapping, and aggregate stability) are governed by the same pore-scale forces. It should be noted that the favorable matching between aggregate size (5–20 μm) and pore-throat size (20–100 μm) in our 2D model may not persist in real reservoirs with broader pore-size distributions; this could affect plugging efficiency and sweep patterns. The observed plugging of high-permeability channels thus offers valid mechanistic insights for controlling heterogeneity in real reservoirs. Future work will validate these behaviors in 3D porous media (e.g., 3D-printed visualization models) to bridge the gap to field applications.

This microsphere–microbial compound system offers a unique combination of physical and biological mechanisms compared with other combined EOR strategies in the literature, such as polymer–microbial^44,52^ or nanoparticle‑enhanced microbial flooding^53^. Its novelty lies in the observed stable agglomeration of surfactant‑modified polyacrylamide nanospheres with indigenous microbes, which significantly enhances shear resistance and long‑term channel‑blocking capability in fractured, medium‑to‑low permeability reservoirs. This distinct synergistic interaction provides more robust macroscopic sweep improvement and microscopic oil mobilization than previously described systems.

We note that this study has not yet quantitatively evaluated retention and plugging propensity arising from aggregate formation. Therefore, future work should systematically vary micromodel geometry and wettability and conduct experiments across a range of pressures and temperatures to quantify the applicability and plugging risk of the composite system under different conditions.

Although flow‑field metrics furnish indirect evidence for persistent channel blocking by the compound system, this study did not perform effluent analyses to directly confirm the persistence of microsphere-microbe aggregates in the flow field. Future work should include pore‑scale visualization, effluent particle and microbial quantification to validate the proposed mechanism, and include broader laboratory screening and field-oriented evaluations to quantify the applicable permeability window and to assess the role of formation-water chemistry.

## Conclusion

The main findings of this study are summarized below:

1. This study successfully constructed a novel microsphere-microbial compound system and elucidated its synergistic mechanisms for improving the recovery factor, verified through microscopic visualization experiments.
2. The compound system demonstrated significant performance, achieving a recovery factor approximately 7.3% higher than the single microbial solution and 4.5% higher than the single microsphere solution, even when utilizing a halved amount of microspheres.
3. The superior advantages of the compound system are attributed to the complementarity of actions across different stages: (I) During the injection stage, microspheres primarily contribute by reducing interfacial tension and improving wettability. (II) During the static incubation stage, microbes continuously generate metabolites (e.g., surfactants) to sustain the oil displacement effect. Concurrently, the aggregation structures formed by both components achieve relatively stable blocking in dominant channels, promoting effective flow redistribution and expanded sweep efficiency.
4. This study is based on two-dimensional visualization and indoor experiments. Physical simulations and field experiments will be performed on three-dimensional heterogeneous reservoirs in the future, and the contribution ratios of chemical and biological actions in the synergistic mechanism will be further quantified to optimize system design and application strategies.

## Data Availability

The data that support the findings of this study are available from the corresponding author upon reasonable request.
